# Reply to Letter to the Editor: Increased Prevalence of Breast and All-cause Cancer in Female Orthopaedic Surgeons

**DOI:** 10.5435/JAAOSGlobal-D-23-00130

**Published:** 2023-09-06

**Authors:** Loretta B. Chou, Brianna Johnson, Lauren M. Shapiro, Stephanie Pun, Lisa K. Cannada, Antonia F. Chen, Lindsey C. Valone, Sara S. Van Nortwick, Amy L. Ladd, Andrea K. Finlay

**Affiliations:** From the Department of Orthopaedic Surgery (Dr. Chou, Dr. Johnson, Dr. Pun, Dr. Ladd), Stanford University (Dr. Chou, Dr. Johnson, Dr. Pun, and Dr. Ladd); the Department of Orthopaedic Surgery, University of California–San Francisco (Dr. Shapiro); the Department of Orthopaedic Surgery, Hughston Clinic (Dr. Cannada); the Department of Orthopaedic Surgery, Harvard Medical School (Dr. Chen); the Department of Orthopaedic Surgery, California Pacific Orthopaedics (Dr. Valone); the Department of Orthopaedic Surgery, Medical University of South Carolina (Dr. Van Nortwick); and the Department of Orthopaedic Surgery, Palo Alto Veterens Association (Dr. Finlay)

We thank Farkouh and colleagues for reading our publication and commenting on the important link between alcohol use and breast cancer prevalence. We agree that alcohol use among female orthopaedic surgeons warrants additional investigation. However, our study was primarily focused on fluoroscopy, polymethyl methacrylate (PMMA), and protective shielding rather than other risk factors of breast cancer that have been identified in other literature. Although binge drinking may be possible in this population, our one-time survey question asked participants “Approximately, how many alcoholic drinks have you consumed in the past 7 days?” We are not able to extrapolate possible binge drinking or long-term alcohol use from this question. Furthermore, the alcohol question used from the California survey asked “About how many drinks did you have on a typical day when you drank alcohol?” and was from a survey conducted more than 10 years before our survey. We do not know whether a similar pattern of alcohol use would be found had the California residents been asked the same alcohol question a decade later. Our study was not designed to test individual risk factors associated with breast cancer prevalence. Studies, such as Chen et al. (2011), cited by Farkouh and colleagues had repeated measures of alcohol use over years and asked about alcohol use in a typical week are better equipped to answers questions about the link between alcohol use and breast cancer prevalence. We briefly noted a few factors in the study, including the lower body mass index and higher rate of never smoking among orthopaedic surgeons. These factors would also benefit from additional investigation.

